# Exploring the Consequences of Crowd Compression Through Physics-Based Simulation

**DOI:** 10.3390/s18124149

**Published:** 2018-11-27

**Authors:** Libo Sun, Norman I. Badler

**Affiliations:** 1 School of Instrument Science and Engineering, Southeast University, Nanjing 210096, China; 2 Department of Computer and Information Science, University of Pennsylvania, Philadelphia, PA 19104, USA

**Keywords:** crowd simulation, physics-based simulation, crushing, compression, local crowd density

## Abstract

Statistical analysis of accidents in recent years shows that crowd crushes have become significant non-combat, non-environmental public disasters. Unlike common accidents such as fires, crowd crushes may occur without obvious external causes, and may arise quickly and unexpectedly in otherwise normal surroundings. We use physics-based simulations to understand the processes and consequences of compressive forces on high density static crowds consisting of up to 400 agents in a restricted space characterized by barriers to free movement. According to empirical observation and experimentation by others, we know that local high packing density is an important factor leading to crowd crushes and consequent injuries. We computationally verify our hypothesis that compressive forces create high local crowd densities which exceed human tolerance. Affected agents may thus be unable to move or escape and will present additional movement obstacles to others. Any high density crowd simulation should therefore take into account these possible negative effects on crowd mobility and behavior. Such physics-based simulations may therefore assist in the design of crowded spaces that could reduce the possibility of crushes and their consequences.

## 1. Introduction

With the increase of large-scale social activities such as sporting events, religious gatherings, and music concerts in recent years, the issue of crowd safety has been gaining considerable attention, since there is a significant potential for injuries and even death. Even with incomplete statistics, 100 accidents with crushes or stampedes occurred between 2006 and 2016, in which 4011 people were killed and more than 6346 people were injured [1]. These disasters have significant social impacts, although there is a relatively low probability that they occur. Crowd crushing and trampling are crucial problems that require understanding and hopefully mitigation.

Generally, there are two main reasons reported for deaths and injuries in a stampede:

(1) Compressional asphyxia due to excessive crushing of the lungs of some victims;
(2) An individual falls down, is trampled by other people, and is unable to stand up again.

Fruin [2] explained, however, that virtually all crowd deaths are the result of compressive asphyxia and not “trampling” that is often reported in the media. As a result, it is very important to investigate the physical characteristics of a crowd when crushing happens.

There are several approaches to exploring crowd disasters: some try to predict the crowd pressures when crushing occurs; some try to model crowd motions at high and extremely high densities where trampling and crushing occur. Relatively little effort has been placed upon the relationship between local crowd density and the physical factors leading to static crowd compression.

In this paper, we present a physics-based approach to simulating the process of crowd compression leading to dangerous crushing densities. According to Fruin [3], people in a crowd may be passively propelled three meters or more when motion shock waves are generated by other crowd behaviors. We model an experimental virtual environment as three rigid, immovable walls and a fourth movable wall. This fourth wall behaves as a piston with respect to the other three. The crowd is dispersed uniformly with a preliminary dense but safe packing within the four-sided environment. By moving the piston wall, we can impose a desired force on the occupants. The agents are subject to physics-based simulation, and we compute the local density experienced by each agent under these circumstances. High computed densities are likely to signal consequent crushing injuries. Populations of 200, 300, and 400 agents are studied to determine the scalability of our approach to new situations. We compare our results with crowd crushing disasters reported in the media to verify that local crowd density is a crucial factor that should be taken into account to help prevent crowd tragedies.

There are two key contributions of this work:

(1) Developing a physics-based approach to simulate the process of crowd compression;
(2) Verification that local crowd density is a critical factor that predicts when crushing, and thus injuries, are likely to occur.

The remainder of this paper is organized as follows: In the next section, we review related work in crowd crushing and examine the role of crowd density in crowd simulation. Section 3 describes the model we used to simulate crowd compression situations. In Section 4, we do experiments to simulate static crowds with 200, 300, and 400 agents when increasingly compressed by the movable piston wall. Section 5 gives the results and analysis of our experiments. Finally, we provide comparisons and discussion in Section 6.

## 2. Related Work

Crowd simulation research covers many tangible aspects of human locomotive behavior such as the realism of the walking motion itself, collision avoidance, navigation, and local interactions between agents. To produce behaviorally interesting agents, crowd simulation takes one of two approaches: microscopic or macroscopic. Microscopic approaches focus on the realism of individual behavior by simulating the perception, memory, planning and emotion of every agent. Some of these features may be minimized or sacrificed to achieve real-time behaviors. Macroscopic approaches, on the other hand, aim to simulate very large crowds, often with fluid-like motion models; thus the behavior of each individual is not as important as long as the overall crowd movement produces realistic emergent behavior.

### 2.1. Research on Crowd Crushing and Stampedes

All crowd simulation systems, either microscopic or macroscopic, lack the specific consideration of simulating compression, crushing, and trampling that may occur in a crowd. Although the social force model can simulate individual agents’ behaviors in a crowd, such as collision avoidance, it fails to adequately describe the causes behind crushing and trampling; these are situations where collision avoidance is an inappropriate model for actual consequences.

Lee and Hughes [4] studied crowd stampedes and developed a rational model for anticipating the motion of human crowds and increasing their safety. In their model, the crowd motion at both high and very high densities of pedestrians, as described by Hughes [5], is based on kinematic wave theory [6]. Wave theory explains many of the observed features of traffic flow and is generalized to pedestrian crowd flows. Furthermore, general rules of crowd behavior are established according to empirical data obtained from official reports produced by the police, coroners, and royal commissions.

Helbing developed the social force model as an analog to the physics of crowd movement. The social force model solves Newton’s equation for each individual agent and maps the interaction between people and obstacles into repulsive interaction, friction forces, dissipation, and fluctuations [7,8]. By simulating the resulting forces, agents demonstrate realistic collision avoidance, pushing behavior, and variable flow rates.

Heigeas [9] believes that there are two kinds of collective phenomena: interpersonal interactions (small group discussion and negotiation, etc.); and global collective phenomena, such as flowing and jamming. Their research focused on the second type of collective human phenomena, called non-deliberative emergent crowd phenomena. They proposed a physically-based particle model to reproduce emergent crowd behaviors. Individuals are modeled as simple disc-like particles on a two-dimensional ground and immovable objects are modeled as sets of fixed particles represented by rectangles. Interactions between individuals are represented by physically-based forces exerted between pairs of particles. In their model, crowd phenomena are viewed as typical cases of collective self-organization, which is one major cause of crowd stampedes.

Henein and White [10] present a swarm force model based on Kirchner’s field-based model [11], in which the space is represented by a discrete grid, and a static field (unchanged) as well as a dynamic field (updated every time step) are adopted to decide which neighboring cell the agent will go to next. They believe that crowd force should be considered to reflect the characteristics of a crowd and, therefore, they integrate a physical force to simulate the effects of injuries and their influence on other nearby agents. In their model, they adopt four general principles for force modelling (i.e., force is directed, propagated, purposeful, and carries consequences) and found that agents who experience excessive force become injured and can no longer move within the model. Although their model takes the interaction forces among the agents into account, the discrete grid limits packing density to one agent per cell. This is an unrealistic constraint when crowd densities become very high.

Teknomo [12] studied the repulsive forces among agents based on the social force model and presented a microscopic pedestrian simulation model. In his model, two kinds of repulsive forces are considered: one pushes the agent away from the closest agent, the other repels all other surrounding agents. The proposed model is verified by comparing simulation results obtained from the microscopic model with real data collected through videos of pedestrian motions. It only considers the relationship between flow, speed, and density, so it does not consider static crowd behaviors.

### 2.2. Research on Crowd Density

Crowd density is an important factor to consider when simulating crowds. Some researchers [13,14] computed the speed of agents according to crowd density. Narain et al. [15] integrated a unilateral incompressibility constraint (UIC) to reflect the relationship between crowd density and preferred velocity. Some researchers [16] use crowd density information to guide a large number of agents along a wide variety of routes instead of the shortest paths. Others [17,18] navigate agents along short routes to the goal based on the principle of least effort, or according to the minimization of actual energy expenditure while simultaneously avoiding congestion (as local density increases, speed decreases), reducing the amount of movement and maintaining the preferred speed for each agent. Best et al. [19] present an approach that combines physiological and psychological principles and effectively generates pedestrian trajectories that adhere to the fundamental diagram of pedestrian movement. Kang and Han [20] also validate their proposed pedestrian model according to the density–speed relation obtained from the fundamental diagram. The fundamental diagram is constructed from observations of real situations and illustrates the inverse relationship between travel speed and density. As a result, realistic crowd behaviors governed by crowd density can be modeled and claim to show better space utilization.

In summary, there is very little work which considers the relationship between static crowd density and crowd crushing. Therefore, we create a model for simulating dense crowd consequences under compressive forces. In this model, we ignore the movement of agents in the crowd and simulate static crowds only.

### 2.3. Research on Multi-Agent Coalition Formation

Cooperation and coordination is one of the fundamental research issues in multi-agent systems, since a single agent is unable to complete some complex tasks. Coalition formation is a proposed approach to solve this problem and has a wide application in distributed artificial intelligence, public safety networks [21] and resource allocation in machine-to-machine communication [22]. Similar to the idea of coalition formation, groups composed of diverse individuals are analyzed and modeled to simulate realistic crowd behaviors. Thalmann [23] proposed a hierarchical model to simulate virtual human crowds in real time, in which groups are the most complex structure that can be controlled with different degrees of autonomy: guided, programmed and autonomous. Guided behaviors are controlled by external users, programmed behaviors are controlled by scripts, and autonomous behaviors are determined by the virtual agent according to its knowledge, belief and intent. Bayazit [24] integrated roadmap-based path planning techniques with flocking techniques and proposes new approaches to simulate four distinct group behaviors: homing, goal searching, traversing narrow areas and shepherding. Collins et al. [25,26,27] have been working on examining how individuals and groups interact with one another and capturing group behaviors in pedestrian evacuation models and simulation. However, none of these approaches is applicable to the consequences of the dominant compressive forces on a large static crowd: goal-directed movement or group coalitions may be powerless to overcome the presence and density of surrounding agents.

## 3. The Compression Model for Crushing Situations

### 3.1. Agent Model

To model an individual human agent, we adopt a three-dimensional cylinder with an ellipse as its cross section for simplicity. According to statistical analysis of measured human body data, including main thickness, main dimension, main width and height of the subjects from Wang [28], the geometric parameters for each represented agent are set and shown in Table 1.

Furthermore, we integrate physical features for each agent so that they can react to the interaction forces between agents, such as pushing and repulsion, which are shown in Table 2. Agents can respond in physically appropriate ways when compressed. This information is used by the “Rigidbody” simulation in Unity3D which considers gravity and rigid collisions to avoid penetration among agents and the walls.

### 3.2. Environment Model

According to Helbing [7], the physical interactions in a packed crowd add up and cause dangerous pressures up to 4450 N·m^−1^. We modeled the environment where the static crowd is situated in a restricted region with four walls. We made three walls immovable and applied the corresponding force based on the estimated pressure of 4450 N·m^−1^ to one movable wall, acting as a piston, to create crowd compression through area reduction. All space within the walls is packed with agents as densely and as uniformly as possible; a hexagonal closest packing on the representative ellipses models this situation. Thus, agents have no space nor exits for escape when the area compression occurs.

## 4. Experiments

### 4.1. Repulsive Force Computation

Each agent would like to keep as equidistant from nearby agents as possible, even when crowd density is very high. We adopt a repulsive force to model this phenomenon. For agent *i*, we first locate every agent *j* whose distance to agent *i* is less than some threshold *D*, then we add the repulsive force whose direction is opposite to the vector subtracting the position of agent *i* from the position of agent *j*, and finally we obtain the total repulsive force *F_r_* for agent *i*, which is given by

(1) $$F_{r}=\sum_{j\in N_{i}}\left(p_{i}-p_{j}\right)\times \frac{C}{w_{ij}}\;$$

where *p_i_* is the position of agent *i*, *p_j_* is the position of agent *j*, *C* is the constant force, *w_ij_* is proportional to the distance between agent *i* and agent *j*, and *N_i_* includes all neighboring agents close to agent *i*.

### 4.2. Local Crowd Density

**Figure 1:**
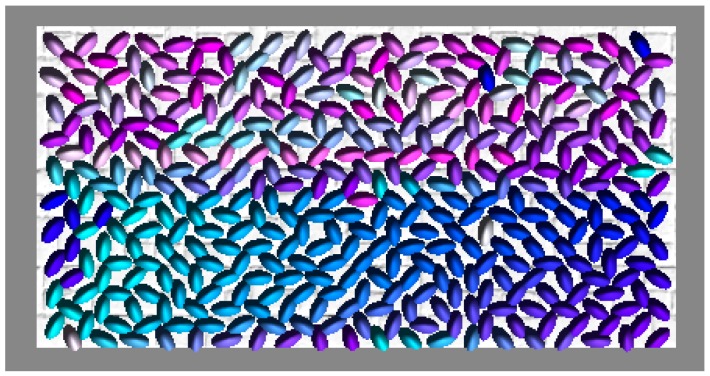
A total of 400 agents are represented with different colors. During simulation the bottom wall moves towards the top wall.

**Figure 2:**
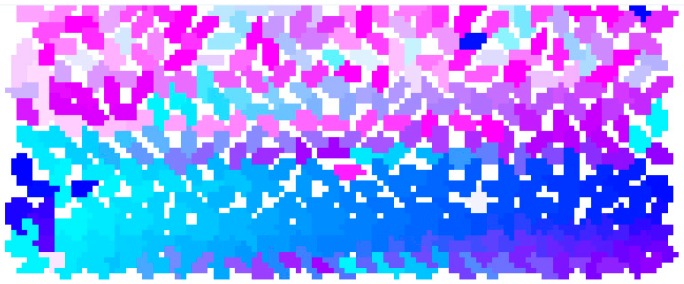
The top view of 400 agents with randomly assigned colors when the bottom wall moves toward the top wall by 1 m.

**Figure 3:**
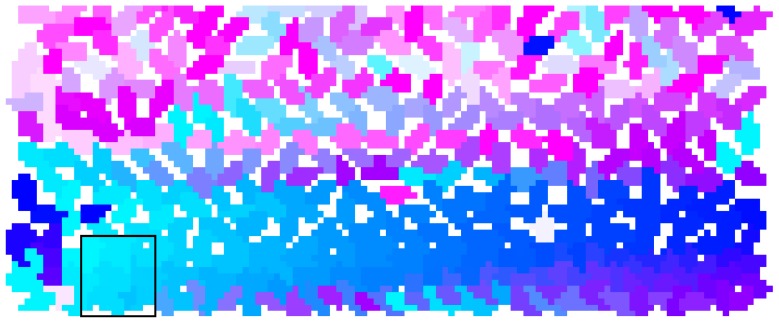
Local crowd density computation. Local crowd density is 10.49 people/m^2^ in the 1 m^2^ area enclosed by the black lines.

**Figure 4:**
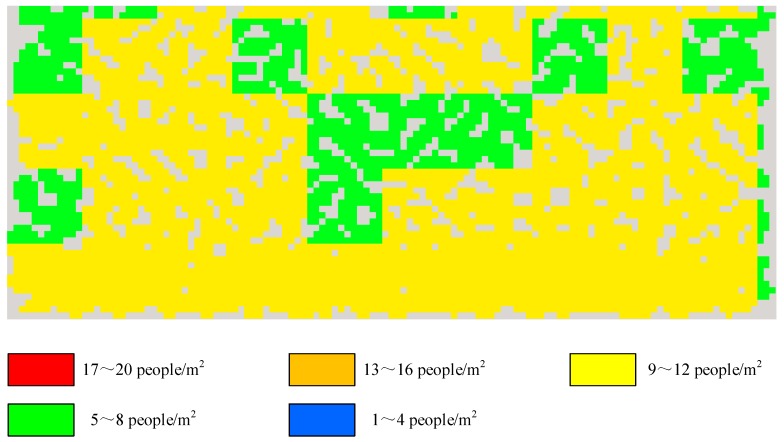
Local crowd density represented by the color scale in Table 3.

We define local crowd density as the number of people per square meter. Inspired by the approaches in [29,30], we computed local crowd density through an image processing algorithm:

(1) We represent each agent’s cylinder with a randomly assigned unique color, which is shown in Figure 1.
(2) The movable wall is translated inward a certain distance to compress the occupied area. During wall movement, Unity3D physics moves the occupants within the space. This step is described in more detail below.
(3) When the wall stops moving, we take a top view image of all agents in the environment, which is shown in Figure 2.
(4) According to the top view image of one red cube whose length and width are both 1 m, we compute the number of pixels which represent one square meter *N_s_*, and correspondingly, we compute the number of pixels which represent one agent *N_a_*.
(5) For each one square meter *S*, we compute the number of pixels *N_c_* for color *c.* Then, the number of agents represented by color *c* is given by
  (2) $$Q_{a}=\frac{N_{c}}{N_{a}}\;$$
  where *Q_a_* is the number of agents *a* in *S*. Thus, local crowd density *ρ_s_* in *S* is given by
  (3) $$\rho_{s}=\sum_{a\in CA}Q_{a}\;$$
  where CA is composed of all agents represented in random colors in S. Figure 3 shows the computation of local crowd density. Note that one square meter starts from the left-bottom corner and then advances 1 m from left to right in sequence until it arrives at the max *X*, then it starts 1 m from the bottom to top and does the same computation until it arrives at the max *Y*. The bottom-left corner is determined by the vertex (min *X*, min *Y*) in which min *X* is the minimum X occupied by some agent while max *X* is the maximum X occupied by some agent, and min *Y* is the minimum Y occupied by a certain agent while max *Y* is the maximum Y occupied by a certain agent.
(6) Finally, we map the local crowd density to a color scale, given in Table 3, and the result is shown in Figure 4.

### 4.3. Static Crowds with 200, 300, and 400 Agents

We seek to discover the relationship between area compression and local crowd density. We conducted three simulations using the Unity3D engine to achieve this goal, based on densely packed populations for 200, 300, and 400 agents. The steps involved are as follows:

(1) We packed 200 agents hexagonally in a rectangular region with length 10.27 m and width 2.76 m; 300 agents hexagonally in a rectangular region with length 10.27 m and width 4.01 m; and 400 agents hexagonally in a rectangular region with length 10.27 m and width 5.28 m. These areas were chosen to maintain a consistent width to the space, and an identical starting density for hexagonal agent packing.
(2) We exerted a force to the one moveable wall to compress the occupied space. The magnitude of the force is determined according to the estimated pressure 4450 N·m^−1^. We made that wall move various distances to reproduce the scenario that “the people in a crowd may be propelled three meters or more when motion shock waves pass through” [3].
(3) We computed local crowd density when the wall moved to each specified distance. For the simulation of the static crowd with 200 agents, we made the wall move inward 0.5 m, 1 m, 1.5 m, and 2 m. For the simulation of the static crowd with 300 agents, we made the wall move inward 0.5 m, 1 m, 1.5 m, 2 m, and 2.5 m. For the simulation of the static crowd with 400 agents, we made the wall move inward 0.5 m, 1 m, 1.5 m, 2 m, 2.5 m, 3 m, and 3.5 m. Computed area compression factors were noted in the following graphs.

## 5. Results

All experiments were run on a laptop with a NVIDIA GeForce GT 730M graphics card and an Intel Core 4 i7-4600U CPU (2.1GHZ) with 8GB memory. Our simulation scenes are constructed using Unity3D version 4.0.0f7.

### 5.1. Simulation Results for a Static Crowd with 200 Agents

**Figure 5:**
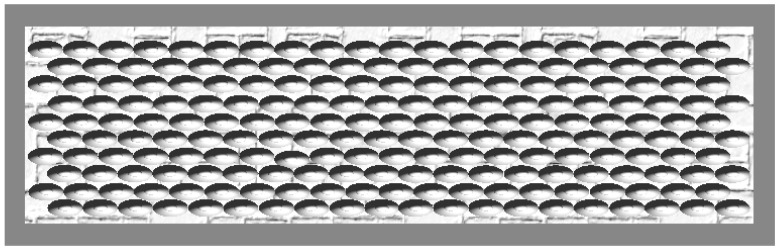
A total of 200 agents are packed hexagonally with three fixed walls, while the wall at the bottom moves upwards in the simulation.

**Figure 6:**
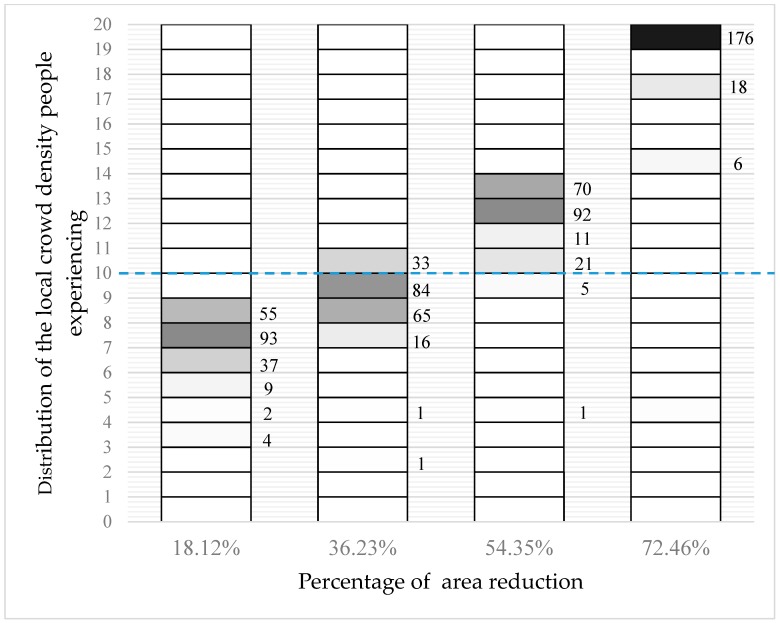
The distribution of local density that the 200 agents experience when the lower wall moves by 0.5 m, 1.0 m, 1.5 m, and 2.0 m to compress the occupied space.

In this scenario, 200 agents are packed hexagonally with 20 agents in each row, which is shown in Figure 5. When the wall at the bottom moves forward 0.5 m, 1.0 m, 1.5 m, and 2.0 m, respectively, the distribution of the local crowd density is shown in Figure 6. Colors encode the number of agents experiencing the indicated density range: white indicates zero or a small number of agents while black indicates a large number of agents. The actual number in each non-zero density range is given to the right of the column. It is apparent that the local crowd density increases continually and faster than linearly when the occupied area shrinks. Ultimately, all agents experience a local crowd density of more than 15 people/m^2^ and, notably, 88% of agents experience a local crowd density of more than 20 people/m^2^. As an approximate guide to a level of injurious density, the blue line shows the 10 people/m^2^ threshold. We can see that more and more people experience a local crowd density above this threshold as the occupied space shrinks.

**Figure 7:**
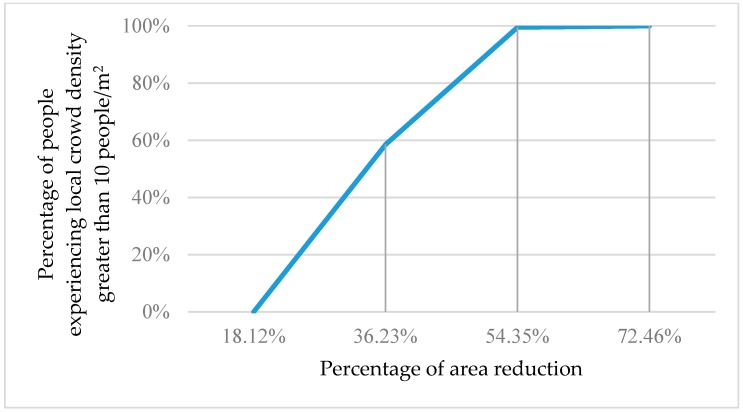
The percentage of 200 people experiencing local crowd density greater than 10 people/m^2^ as the occupied area shrinks.

Figure 7 shows the percentage of people experiencing local crowd density greater than 10 people/m^2^ as the occupied area shrinks.

### 5.2. Simulation Results for Static Crowd with 300 Agents

**Figure 8:**
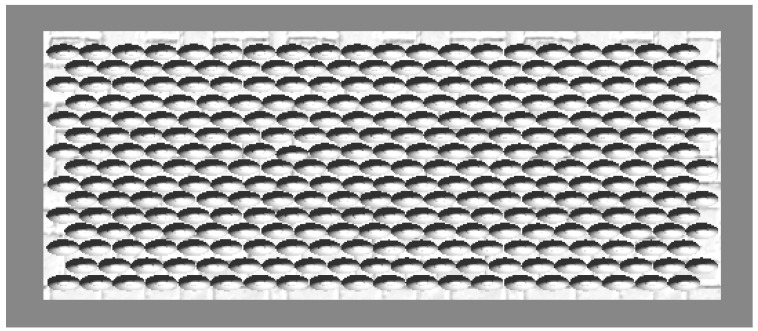
A total of 300 agents are packed hexagonally with three fixed walls, while the wall at the bottom moves upwards in the simulation.

**Figure 9:**
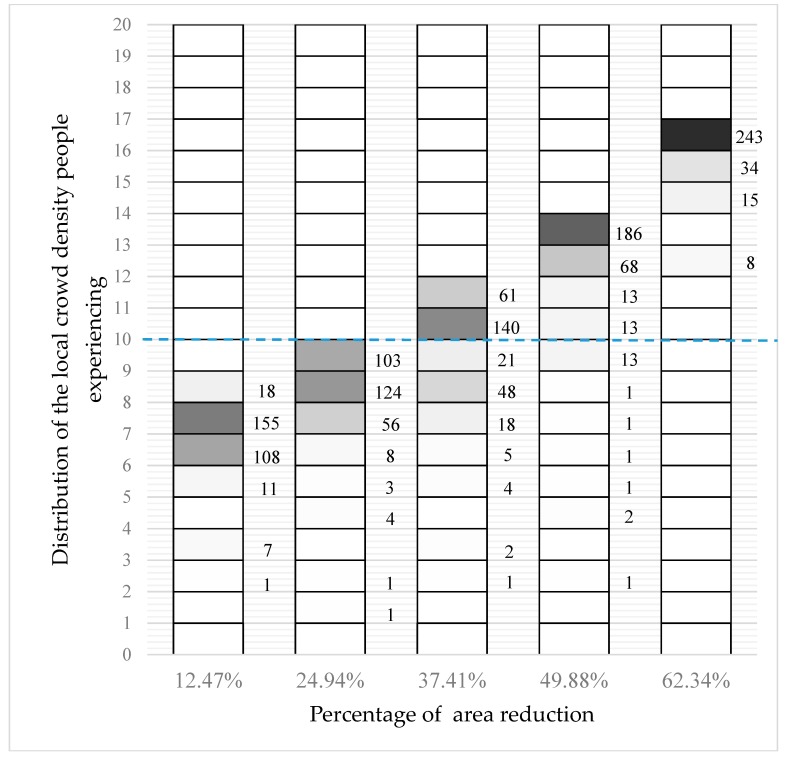
The distribution of local density that 300 agents experience when the lower wall moves by 0.5 m, 1.0 m, 1.5 m, 2.0 m, and 2.5 m to compress the occupied space.

In this scenario, 300 agents are packed hexagonally with 20 agents in each row, which is shown in Figure 8. When the wall at the bottom moves forward at 0.5 m, 1.0 m, 1.5 m, 2.0 m, and 2.5 m, respectively, the distribution of the local crowd density is shown in Figure 9, with the same format and interpretation as above. It can be seen that the local crowd density increases continually when the occupied area shrinks and, finally, all agents experience a local crowd density of more than 13 people/m^2^, while 97.3% of agents experience a local crowd density of more than 15 people/m^2^.

**Figure 10:**
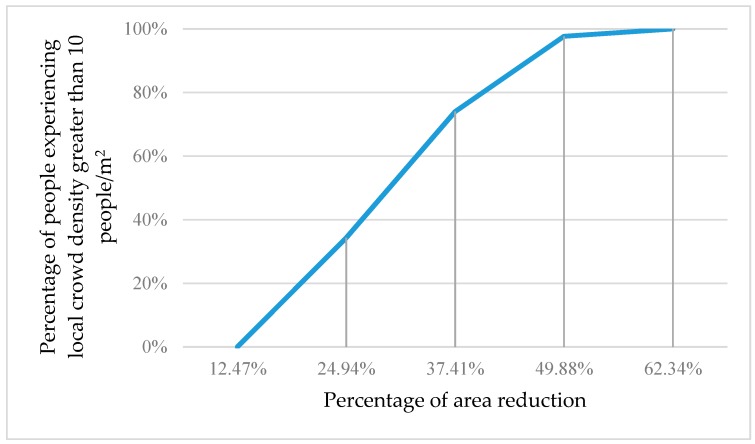
The percentage of people experiencing local crowd density greater than 10 people/m^2^ as the occupied area shrinks.

Figure 10 shows the percentage of people experiencing a local crowd density greater than 10 people/m^2^ as the occupied area shrinks.

### 5.3. Simulation Results for Static Crowd with 400 Agents

**Figure 11:**
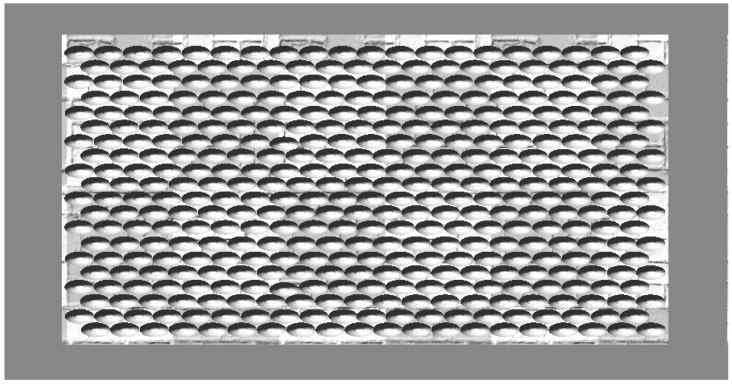
A total of 400 agents are packed hexagonally with three fixed walls, while the wall at the bottom moves upward in the simulation.

**Figure 12:**
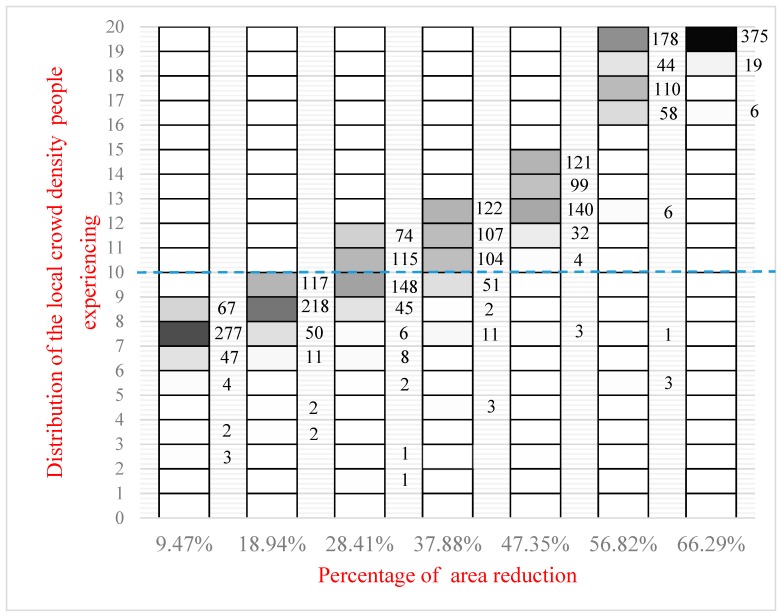
The distribution of local density that 400 agents experience when the lower wall moves by 0.5 m, 1.0 m, 1.5 m, 2.0 m, 2.5 m, 3.0 m, and 3.5 m to compress the occupied space.

**Figure 13:**
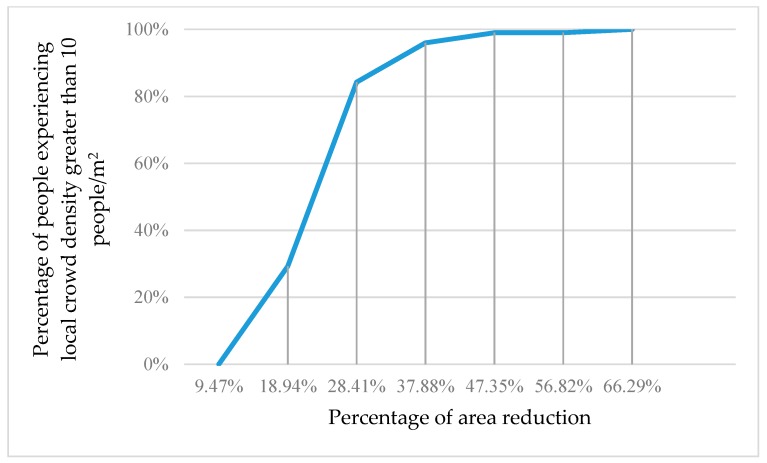
The percentage of people experiencing local crowd density greater than 10 people/m^2^ as the occupied area shrinks.

In this scenario, 400 agents are packed hexagonally with 20 agents in each row, which is shown in Figure 11. When the walls moves forward at 0.5 m, 1.0 m, 1.5 m, 2.0 m, 2.5 m, 3.0 m, and 3.5 m, respectively, the distribution of the local crowd density is shown in Figure 12, with the same format and interpretation as above. It can be seen that local crowd density increases continually when the occupied area shrinks and finally, all agents experience a local crowd density of more than 17 people/m^2^, while 93.75% of agents experience a local crowd density more than 20 people/m^2^. Figure 13 shows the percentage of people experiencing a local crowd density greater than 10 people/m^2^ as the occupied area shrinks.

**Figure 14:**
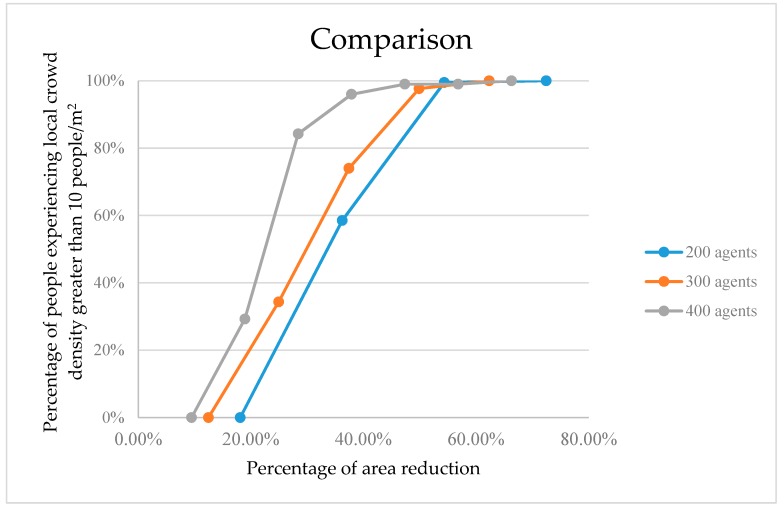
The percentage of people experiencing local crowd density greater than 10 people/m^2^ as the occupied area shrinks for the three population sizes.

Figure 14 compares the percentage of people experiencing a local crowd density greater than 10 people/m^2^ across all three simulation cases. It can be seen that when the occupied area containing a static crowd with 400 agents shrinks by about 20%, the percentage of people experiencing a local crowd density greater than 10 people/m^2^ increases to almost 60%.

From Figure 14, it appears that as crowd size increases, the percentage of individuals experiencing unsafe high densities increases, even at just a 25% reduction in occupied area. With a 50% area reduction, virtually all crowd individuals are fully compromised. Our physics-based approach to computing compression stress on densely packed crowds thus appears consistent and scalable to larger crowds, and may provide another predictive tool to evaluate potential dangers in obstructive environments.

## 6. Comparison and Discussion

We use physics-based simulations to understand the processes and consequences of compressive forces on high density static crowds consisting of up to 400 agents in a restricted space characterized by barriers to free movement. We computed the local density experienced by each individual and showed that human injury may occur with as little as a 20% reduction in area, and that densities become dangerously high for crowds of 200 to 400 people when the occupied area shrinks by 25%. Compared with social force models [7], which allow for realistic pushing behavior and variable flow rates, our approach is physics-based and focuses on local crowd density within a rigid barrier geometric model. We used an elliptical cross-section human body surrogate that allowed us to begin the simulation with a reasonably dense crowd packing, but one that was still within the safe range. We opted to use cylinders without movable arms, as the principal danger in crowd crushes is torso compression and the consequent inability to breathe. The ellipsoid models the presence of the shoulder within the torso cross-section and this shape yields denser, less uniform packing at low compression, but quickly offers no “wiggle room” as compression increases. Furthermore, social force models are unsuitable for high density crowd simulation, since there is expected space between individuals from the repulsive force and it is thus difficult to deal with crowd densities greater than 4 people/m^2^ [31]. Cellular automata models [32] are defined as mathematical idealizations of physical systems in which space and time are discrete, and physical quantities take a finite set of discrete values. The cellular grid defines the closest possible agent packing. Rule-based models [23,33] describe human movement with a set of rules and achieve more realistic human movement for low and medium density crowds, but cannot handle contact between individuals and therefore fail to simulate pushing and crushing behaviors. In Table 4, we summarize how these models cannot deal with the simulation of high density crowd and how our approach can simulate high density crowds with local agent densities greater than 10 people/m^2^.

By using the physics simulation in Unity3D, we achieved real-time simulations. The evolution of the crowd crush situation can be observed directly while the piston-like wall compresses the crowd. Other physics-based simulators in other animation platforms can be readily substituted. To build a valid physical simulation we used data we found reported in the media. Our results illustrate that local crowd density is a crucial factor to be considered for the prevention of crowd crush disasters.

## Figures and Tables

**Table 1:** Agent parameters.

Attributes	Value
Height	1.7 m
Horizontal width	0.5 m
Vertical width	0.25 m
Orientation	Random
Packing pattern	Hexagonal

**Table 2:** Physical Parameters of Agents.

Attributes	Value
Mass	50 kg
Drag	0
Angular Drag	0.05
Use gravity	Yes
Collider	Capsule Collider

**Table 3:** Mapping of local crowd density and color scale.

Local crowd density	Color scale
1~4	blue
5~8	green
9~12	yellow
13~16	orange
17~20	red

**Table 4:** Comparisons with classical crowd simulation models.

	Space	Physics-Based	Crowd Density
Social force model	continuous	No	Difficult to handle crowd with crowd density greater than 4 people/m^2^
Cellular automata model	discrete	No	Low; limited by grid resolution
Rule-based model	continuous	No	Low or medium
Our approach	continuous	Yes	Can handle crowd with crowd density greater than 10 people/m^2^
